# Alternol Sensitizes Renal Carcinoma Cells to TRAIL-Induced Apoptosis

**DOI:** 10.3389/fphar.2021.560903

**Published:** 2021-03-25

**Authors:** Yu Ren, Xue Wang, Shuaishuai Huang, Yangkai Xu, Guobin Weng, Rui Yu

**Affiliations:** ^1^ Department of Urologic Surgery, Ningbo Urology and Nephrology Hospital, Ningbo Yinzhou No 2. Hospital, Ningbo, China; ^2^ Department of Biochemistry and Molecular Biology, Zhejiang Provincial Key Laboratory of Pathophysiology, School of Medical, Ningbo University, Ningbo, China

**Keywords:** tumor necrosis factor–related apoptosis-inducing ligand, alternol, reactive oxygen species, apoptosis, renal carcinoma cancer

## Abstract

**Purpose:** Tumor necrosis factor–related apoptosis-inducing ligand (TRAIL), a member of the TNF family, can selectively induce cancer cell death while sparing normal cells. However, the application of TRAIL-based antitumor therapies has been hindered due to drug resistance. Alternol is a new compound isolated from microbial fermentation that possesses antitumor activity in different tumors. In our research, we discovered that alternol can sensitize TRAIL-induced apoptosis in renal carcinoma cells (RCCs).

**Materials and Methods:** Cytotoxic activity was measured by MTT assay. Apoptosis was probed using the PI/annexin V method. Real-time PCR and western blot were used to test the levels of mRNA and protein, respectively. Luciferase assay was used to investigate whether CHOP regulated the expression of death receptor (DR) 5 through transcription. A xenogeneic tumor transplantation model was used to evaluate the anticancer effects of alternol/TRAIL *in vivo*.

**Results:** When the mechanisms were investigated, we discovered that alternol increased DR5 expression. DR5 knockdown by siRNA eliminated the enhanced effect of alternol on TRAIL-mediated apoptosis. Alternol reduced the expression of antiapoptotic proteins and increased the levels of proapoptotic proteins. Moreover, alternol increased the level of CHOP, which is necessary for the enhancing effect of alternol on TRAIL-induced apoptosis, given that downregulation of CHOP abrogated the synergistic effect. DR5 upregulation induced by alternol required the production of reactive oxygen species (ROS). Removing ROS inhibited the induction of DR5 and blocked the antiapoptotic proteins induced by alternol.

**Conclusion:** Taken together, our research suggested that alternol increased TRAIL-mediated apoptosis via inhibiting antiapoptotic proteins and upregulating DR5 levels via ROS generation and the CHOP pathway.

## Introduction

Tumor necrosis factor–related apoptosis-inducing ligand (TRAIL) is a member of the TNF super family. TRAIL is a promising antitumor agent, given that it selectively induces apoptosis in tumor cells while sparing normal cells (Ashkenazi, 2015). Currently, five different TRAIL receptors have been identified. Among them, death receptor (DR) 4 (DR4, TRAIL-R1) and DR5 (TRAIL-R2) are functional receptors. Other TRAIL receptors, such as decoy receptor-1 and -2 (DcR1 and DcR2) and osteoprotegerin (OPG), exhibit dominant negative effects through competing with DR4 and DR5 for TRAIL binding (Mahalingam et al., 2009). After binding to DR4 and/or DR5, TRAIL recruits Fas-associated death domain (FADD) and procaspase-8 into a death-inducing signaling complex (DISC) (Lemke et al., 2014). The autocatalytic processing of procaspase-8 into caspase-8 within the DISC leads to the subsequent downstream activation of the executioner caspase-3.

Given that many human carcinoma cells evade TRAIL-induced apoptosis, unveiling and overcoming the mechanisms behind this resistance are necessary. In cells, the process of TRAIL-induced apoptosis is regulated by various intracellular mechanisms in addition to the DR expression levels, such as the expression of Bcl-2 family members, which can be further divided into proapoptotic and antiapoptotic members. Moreover, cellular FLICE-inhibiting protein (FLIP) and IAP family members, such as XIAP, IAP-1, and IAP-2, also affect TRAIL-induced apoptosis. FLIP functions as a caspase-8 inhibitor, whereas XIAP directly binds to caspase-9 and -3 to inhibit their activation (Ashkenazi, 2008). IAP-1 and IAP-2 trigger RIP1 ubiquitination at the DR complex level, thereby interfering with apoptosis initiation (Mahoney et al., 2008). Accordingly, agents that inhibit such antiapoptotic proteins and/or upregulate proapoptotic proteins can potentially sensitize carcinoma cells to TRAIL-induced apoptosis (Prasad et al., 2014).

Alternol is a novel compound purified from the fermentation product of the novel microorganism mutant strain *Alternaria alternata* var. *monosporus*, which was obtained from the bark of yew trees in Kunming, southwest of China (Liu et al., 2007). Alternol can trigger cell cycle arrest and apoptosis as well as block proliferation and the epithelial-to-mesenchymal transition in tumor cells (Tang et al., 2014). Moreover, a recent study indicated that alternol selectively kills prostate cancer cells but not normal prostatic epithelial cells (Yeung et al., 2012). Therefore, we were interested in testing the effect of alternol and TRAIL cotreatment in carcinomas.

In the current study, we examined whether alternol can potentiate TRAIL-induced apoptosis in human renal carcinoma cells (RCC) *in vitro* and in a tumor xenograft mouse model. The mechanisms by which alternol might enhance TRAIL activity were also investigated. Our results revealed that the combined alternol and TRAIL treatment may facilitate the development of an effective cancer therapeutic strategy.

## Materials and Methods

### Reagents

Alternol was purchased from Strand Biotech Co., (Shantou, China). Alternol was dissolved in DMSO at the concentration of 1 mM and kept at −20°C. Recombinant human TRAIL was provided from Life Technologies (Frederick, MD). Antibodies specific for DR5 (cat no 3696; dilution 1:1,000), DR4 (cat no 42533; dilution 1:1,000), Bax (cat no 8023; dilution 1:1,000), caspase-8 (cat no 9746; dilution 1:1,000), caspase-3 (cat no 9662; dilution 1:1,000), phospho-Akt (Ser 473) (cat no 4060; dilution 1:1,000), phospho-ERK (cat no 4337; dilution 1:1,000), total Akt (cat no 4658; dilution 1:1,000), and ERK (cat no 4696; dilution 1:1,000) were purchased from Cell Signaling Technology (Danvers, MA). Phycoerythrin (PE)-conjugated antibodies for DR4 (cat no FAB347P), DR5 (cat no FAB6311P), DcR1 (cat no FAB6302P), DcR2 (cat no FAB633P), and human IgGs (cat no 1-001-A) were obtained from R&D Systems (Minneapolis, MN). Antibodies against phospho-JNK (Thr183/185) (cat no sc-293136; dilution 1:1,000), JNK (cat no sc-7345; dilution 1:1,000), and cytochrome c (cat no sc-48432; dilution 1:1,000) were obtained from Santa Cruz Biotechnology (Santa Cruz, CA). Antibodies against XIAP (cat no AF8221; dilution 1:1,000) and survivin (cat no AF886; dilution 1:1,000) were purchased from R&D Systems (Minneapolis, MN). Antibodies against Bcl-2 (cat no ab32124; dilution 1:1,000), Mcl-1 (cat no ab32087; dilution 1:1,000), c-IAP1 (cat no ab108361; dilution 1:1,000), c-IAP2 (cat no ab32059; dilution 1:1,000), Bcl-xl (cat no ab32370; dilution 1:1,000), PPARγ (cat no ab178860; dilution 1:1,000), and CHOP (cat no ab10444; dilution 1:1,000) were obtained from Abcam (Cambridge, MA). The antibody against GAPDH (cat no G5262; dilution 1:5,000) was obtained from Sigma (St. Louis, MO, United States). Penicillin, streptomycin, RPMI 1640, fetal bovine serum, and dichlorofluorescein diacetate (DCF-DA) were purchased from Life Technologies (Frederick, MD). An annexin V/PI binding kit was purchased from BD Biosciences (San Jose, CA). All other chemicals were obtained from Sigma (St. Louis, MO).

### Cell Culture

The human renal carcinoma Caki-1, ACHN, and A498 cell lines were obtained from the Shanghai Cell Resource Center (Shanghai, China). Fresh RCC cells derived from four patients with RCC were isolated from surgical specimens as described previously (Wu et al., 2000). The histological diagnosis showed that all patients had RCC of the alveolar type and a clear cell subtype. Normal kidney cells from the same patients were isolated from the normal tissues, which were separated clearly from tumor tissues as described previously (Wilson et al., 1985). The normal kidney cells expressed the epithelial membrane antigen, confirming that the cells were of epithelial origin. All normal kidney cells were able to survive to the third or fourth generation only. Cells were cultured in RPMI 1640 media containing 10% fetal bovine serum and 1% penicillin/streptomycin and were incubated at 37°C in a humidified chamber with 5% (v/v) CO_2_. Written informed consent forms were obtained from participants. The approval of this study was endowed by the Ethics Committee of Ningbo Yinzhou No. 2 Hospital.

### Cytotoxicity Assay

The MTT assay was applied to measure the RCC cytotoxic activity of alternol and TRAIL individually and in combination. After the drug treatment, the cells were incubated with 0.5 mg/ml MTT at 37°C for 3 h. The MTT product was solubilized with dimethyl sulfoxide (DMSO) and measured at 570 nm using a BioTek plate reader (Winooski, VT, United States).

### Caspase-3/-8 Activity Assay

Caspase-3 and -8 activity were determined using a caspase-3 and -8 multiplex activity assay kit (Fluorometric) (cat no ab219915; Abcam; Cambridge, United States). Briefly, after different treatments, cells were washed with PBS three times and added lysis buffer and then recentrifuged for 5 min at 14,000 g. Supernatant samples were used to measure caspase-3 and -8 activity, and protein concentrations were measured using the Bradford method. Then, 50 μl of supernatant with 10 μl (2 nM) of caspase-3 and -8 substrate acetyl-Asp-Glu-Val-Asp p-nitroanilide (Ac-DEVD-pNA) and acetyl-Ile-Glu-Thr-Asp (Ac-ITED-pNA) was incubated at 37°C for 2 h. Finally, the absorbance of yellow pNA was calculated with a spectrometer at 405 nm.

### Flow Cytometry

The apoptotic rate was measured using an annexin V/PI assay and then analyzed with a flow cytometer (FACSCalibur, BD Biosciences). To measure receptor expression, a total of 1 × 10^6^ cells was resuspended in PBS and then incubated with PE-conjugated antibodies at room temperature for 0.5 h. human IgGs with PE were used as isotype controls. Cell fluorescence intensity was measured by flow cytometry (FACSCalibur, BD Biosciences), and the results were analyzed using FlowJo software. To measure intracellular reactive oxygen species (ROS) generation, the cells were pretreated with dichlorofluorescein diacetate (DCF-DA) (Sigma) at 37°C for 10 min before incubating with alternol for an additional 6 h. Cells were then harvested and washed with PBS, and the fluorescence intensity of intracellular DCF (excitation 488 nm, emission 530 nm) was monitored using a flow cytometer (FACSCalibur, BD Biosciences).

### RNA Isolation and Real-Time PCR

Total RNA was purified from RCC cells using TRIzol reagent (Life Technologies; Carlsbad, CA, United States) according to the manufacturer’s guide. Total RNA was reversed transcribed into cDNA using the PrimerScriptTM RT reagent kit (TaKaRa, Dalian, China). The reaction was performed on the ABI 7500-Fast qRT-PCR System. The specific primers used for real-time PCR were as follows: actin (forward) 5′-CGC​CCT​AGG​CAC​CAG​GGT​GTG-3′ and (reverse) 5′-TCG​GTG​AGC​AGC​ACA​GGG​TG-3′; DR4 (forward) 5′-CGA​TGT​GGT​CAG​AGC​TGG​TA-3′ and (reverse) 5′-ACG​GCA​GAG​CCT​GTG​CCA​TC-3′; DR5 (forward) 5′-GGG​AGC​CGC​TCA​TGA​GGA​AG-3′ and (reverse) 5′-AGT​CTC​TCT​CCC​AGC​GTC​TC-3′; DcR1 (forward) 5′-CCC​AAA​GAC​CCT​AAA​GTT​CGT​C-3′ and (reverse) 5′-GCA​AGA​AGG​TTC​ATT​GTT​GGA-3′; and DcR2 (forward) 5′-ACC​CCA​AGA​TCC​TTA​AGT​TCG-3′ and (reverse) 5′-CAA​GAA​GGC​AAA​TTG​TTG​GAA-3′. Actin expression served as an internal control. The relative expression was assessed using the comparative 2−ΔΔCt method.

### Luciferase Assay

The following plasmids were obtained from Professor Chenguo Yao (Zhejiang University, Hangzhou, China) as gifts: pDR-2500 [containing DR5 promoter sequence (−2,500/+30)], pDR-605wt [containing DR5 promoter sequence (−605/+3)], and pDR-605mut [containing mutated DR5 promoter sequence (−605/+3)]. The activities of luciferase and β-galactosidase were measured using the Dual-Luciferase Reporter Assay System (Promega, Madison, WI, United States) according to the manufacturer’s protocol. Luciferase activity was normalized to β-galactosidase activity, and three independent experiments were performed.

### Western Blot Assay

Cells were collected and lysed on ice using RIPA lysis buffer (Beyotime, Beijing, China) containing cocktail protease inhibitors (Sigma, St. Louis, MO). The protein concentrations were assayed with a Bradford protein assay kit (Beyotime). Then, 20 µg of proteins were loaded and subjected to 12% SDS-PAGE and were subsequently transferred to PVDF membranes (Thermo Fisher Scientific, Inc.). The proteins transferred to the membranes were biologically reacted with primary antibody at the recommended dilutions followed by incubations with the corresponding secondary antibodies 12 h later. Ultimately, the results were visualized using an enhanced chemiluminescence (ECL) kit (Thermo, Roalternolford, CA, United States).

### RNA Interference

siRNA against DR4, DR5, PPARγ and CHOP, and scramble siRNA were synthesized and purchased from GenePharma (Suzhou, Jiangsu, China). Transfection was performed using the Lipofectamine 2000 (Life Technologies) according to the manufacturer’s guide.

### MDA and CAT Activity Assay

Cells were washed twice with cold PBS, and total proteins were extracted using 100 μl RIPA lysis buffer. Lysates were centrifuged at 13,000 g for 5 min. The supernatants were quantified using Bradford protein assay kit (Beyotime, Beijing, China). MDA equivalents and CAT levels were measured with a commercially available kit according to the manufacturer’s instructions (Beyotime, Beijing, China).

### Tumor Xenograft Model

Six- to four-week-old male nude mice were obtained from the Animal Center of School of Medicine, Ningbo University (Ningbo, China). The animal study was approved by the Ethical Committee on Animal Research of Ningbo University. Cells were harvested and suspended in RPMI 1640 medium. Tumors were induced by injecting 5 × 10^6^ cells mixed with Matrigel (1:1) (Sigma-Aldrich) into the right side of the mice. It took around 10 days for tumor size to reach approximately 3–5 mm in diameter; the mice were divided into four groups (eight to nine mice per group). The mice were treated with an intravenous injection of saline (control) or alternol (20 mg/kg) or TRAIL (3 mg/kg) or a combination of alternol and TRAIL. The tumor sizes were measured with calipers every three days, and two perpendicular diameters of tumors were recorded. The mice were sacrificed, and the tumors were resected and weighed at the end of the study.

### Statistical Analysis

SPSS software (version 12; SPSS Inc., Chicago, IL, United States) was employed to perform statistical analyses. The data were presented as the means ± standard deviations (SD). One-way ANOVA was performed to measure the differences between groups. A significant difference was considered at *p* < 0.05.

## Results

### Alternol Increased TRAIL-Induced Apoptosis in Renal Carcinoma Cells

**FIGURE 1:**
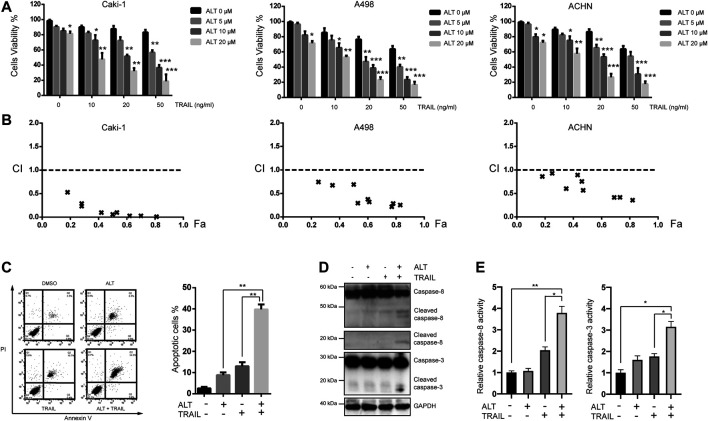
Alternol sensitizes renal cancer cells to TRAIL. **(A)** Renal carcinoma cell lines Caki-1, A498, and ACHN were treated with various concentrations of alternol (ALT) (0, 5, 10, nad 20 μM) and TRAIL (10, 20, and 50 ng/ml) for 24 h. Cell viability was assessed by the MTT assay. **(B)** Combination index (CI) values with fraction affected (Fa) between alternol and TRAIL in Caki-1, A498, and ACHN cells were calculated using the CalcuSyn software. **(C)** Caki-1 cells were treated with TRAIL (50 ng/ml), alternol (20 μM) alone or in combination for 24 h. Cells were stained with PI/annexin V and analyzed by FACS. Quantitative analysis of cell death rate upon treatments described above. **(D)** Whole-cell extracts were subjected to western blot assay using indicated antibodies. **(E)** Caki-1 cells were treated with TRAIL (50 ng/ml) and alternol (20 μM) alone or in combination for 24 h. Then, caspase-3/-8 activities were assayed. Data are the mean ± SD of three independent experiments. *p < 0.05; **p < 0.01; ***p < 0.001, compared with 0 μM ALT.

We examined the effect of alternol on various RCCs. Caki-1, A498, and ACHN cells were treated with various alternol concentrations (0, 5, 10, and 20 μM) alone for 24 h, and cell viability was assessed using MTT assay. The viability of these cells was inhibited by alternol in a dose-dependent manner (Figure 1A). Furthermore, we examined the sensitivity of these cell lines to TRAIL treatment. These cells were treated with different doses (0, 10, 20, and 50 ng/ml) of TRAIL for 24 h. These three RCC lines were moderately sensitive to TRAIL treatment (Figure 1A). Meanwhile, cotreating RCCs with alternol significantly increased the TRAIL-induced cytotoxicity in a dose-dependent manner (Figure 1A). To examine whether this synergy reflected properties of established tumor cell lines, we also tested for synergy in four samples of freshly isolated RCCs. As indicated in Supplementary Figure S1, significant synergy was achieved in all cases. However, the alternol and TRAIL combination failed to induce synergistic cytotoxicity in freshly isolated normal kidney cells (Supplementary Figure S2).

Given that Caki-1 cells were less sensitive to TRAIL than the other two cell lines, we chose to investigate whether the synergistic cytotoxicity of TRAIL and alternol cotreatment in Caki-1 cells was mediated by apoptosis. Annexin V/PI assay was performed to measure the apoptotic cells. Early and late apoptotic cells are presented in the upper-right and lower-right quadrant, respectively. We found that 20 μM alternol or 50 ng/ml TRAIL alone induced up to 9 and 15% apoptosis, respectively. However, when cells were cotreated with alternol and TRAIL, the apoptosis rate was increased to greater than 40% (Figures 1B,C). To further investigate the effect of alternol on TRAIL-induced apoptosis, we examined caspase activation after treatment for 24 h, which is a hallmark of apoptosis. As shown in Figure 1D, TRAIL alone had a minimal effect, whereas alternol sensitized TRAIL-induced caspase-8 and -3 activation. Moreover, caspase-3/-8 activity assays also showed that combination of alternol and TRAIL significantly increased the activities of caspase-3/-8 compared with treatment with alternol or TRAIL alone (Figure 1E). These results indicate that alternol enhances TRAIL-induced apoptosis and caspase activation in RCCs.

### Alternol Increases DR5 Expression Independent of p53

**FIGURE 2:**
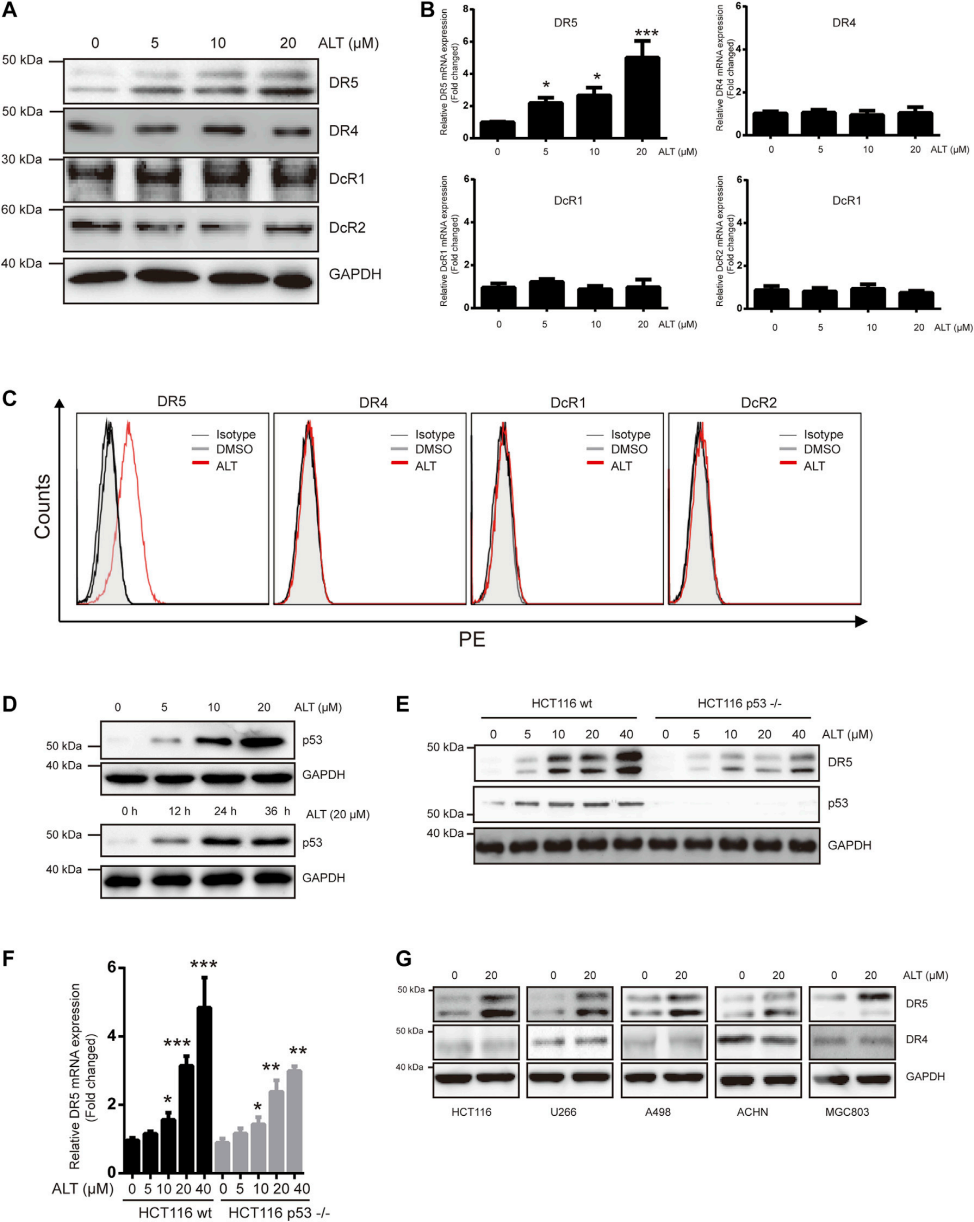
Alternol induces expression of DR5. **(A)** Caki-1 cells were treated with various doses of alternol (ALT) for 24 h. Total cellular extracts were then prepared and analyzed for DR4, DR5, DcR1, and DcR2 by western blot. **(B)** Caki-1 cells were treated with various doses of alternol (ALT) for 24 h. DR5, DR4, DcR1, and DcR2 mRNA levels were analyzed by qRT-PCR. **(C)** Cellular surface levels of DR5, DR4, DcR1, and DcR2 were analyzed by flow cytometry staining using PE-conjugated specific antibodies for each receptor. IgG isotype controls (gray histogram), DMSO (gray line), and alternol (red line) are presented. **(D)** Caki-1 cells were treated with various doses of alternol for 24 h or treated with 20 μM alternol for different times. Then, cellular lysates were analyzed with indicated antibodies. HCT116 wt and HCT116 p53−/− cells were treated with various doses of alternol for 24 h, and DR5 protein and mRNA levels were analyzed by western blot **(E)** and qRT-PCR **(F)**, respectively. **(G)** Various types of carcinoma cells were treated with 20 μM alternol for 24 h, after which whole-cell extracts were analyzed by western blot assay. The same blots were stripped and re-incubated with actin antibody to confirm equal protein loading. Data are the mean ± SD of three independent experiments. *p < 0.05; **p < 0.01; ***p < 0.001, compared with 0 μM ALT.

TRAIL induces and mediates apoptotic activity mainly through DR4 and/or DR5. Therefore, we tested whether alternol sensitized TRAIL-induced apoptosis through modulating DR4 and/or DR5 expression. Treating Caki-1 cells with different concentrations of alternol for 24 h resulted in a noticeable dose-dependent upregulation in DR5 levels but not DR4 levels (Figure 2A). The decoy receptors DcR1 and DcR2 compete with functional DR for TRAIL binding and thereby interfere with TRAIL-induced apoptosis (Gupta et al., 2011). Therefore, we also examined whether alternol affects DcR expression. We found that alternol did not influence DcR1 or DcR2 levels (Figure 2A). Then, we used qRT-PCR to measure DR4, DR5, DcR1, and DcR2 mRNA levels after alternol treatment for 12 h. Consistent with the western blot results, DR5 mRNA levels were upregulated by alternol in a dose-dependent manner (Figure 2B). However, DR4, DcR1, and DcR2 mRNA levels were not affected (Figure 2B). In addition, we also examined whether alternol affects the expression of DRs and DcRs on the cell surface. As expected, we found that alternol increased the cell surface level of DR5 only; the levels of DR4, DcR1, and DcR2 were not changed (Figure 2C). An increasing amount of evidence indicates that p53 induces DR5 upregulation (Tomasetti et al., 2006). We asked whether alternol can induce p53, and, if so, whether this effect mediates DR5 induction. As shown in Figure 2D, alternol induced p53 up-regulation in a dose- and time-dependent manner in Caki-1 cells. To examine whether p53 is required for alternol-induced DR5 induction, we used HCT116 p53−/− cells, in which p53 is knocked out. As indicated in Figures 2E,F, however, alternol upregulated DR5 protein and mRNA levels in both wild-type and p53-deleted cells. However, the extent of upregulation of DR5 was attenuated in p53-deleted cells. These findings suggested that alternol-induced upregulation of DR5 at least not entirely relied on the p53. Finally, we also investigated whether alternol-induced DR5 upregulation was specific to Caki-1 cells. We treated the following cells with alternol for 24 h: H1299, U266, A498, ACHM, and MGC803 cells. Alternol induced the expression of DR5 but not DR4 in all these cell lines (Figure 2G). These results suggest that the DR5 expression induced by alternol is not cell-type specific.

### Alternol-Induced DR5 is Essential for TRAIL-Induced Apoptosis

**FIGURE 3:**
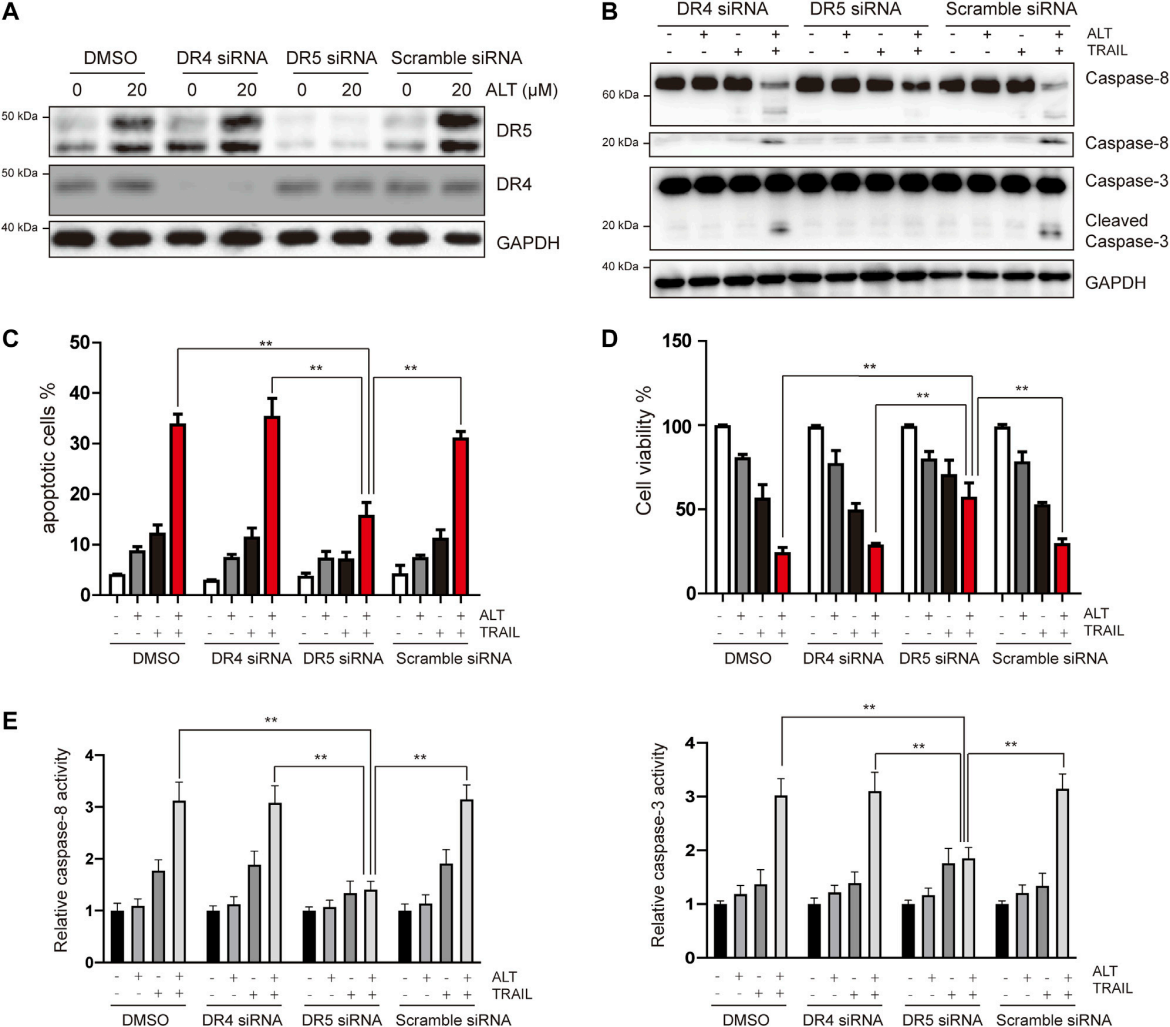
Upregulation of DR5 is essential for alternol-induced sensitization of TRAIL. **(A)** Caki-1 cells were transfected with DR5 siRNA, DR4 siRNA, or scramble siRNA. After 24 h, cells were treated with 20 μM alternol for an additional 24 h, and total cellular extracts were subjected to western blot assay using indicated antibodies. **(B)** Caki-1 cells were transfected with DR5 siRNA, DR4 siRNA, or scramble siRNA for 24 h, then cells were treated with alternol (20 μM), TRAIL (50 ng/ml), or combination for another 24 h, and then cellular lysates were subjected to western blot with indicated antibodies. **(C)** Cells were treated as described as above, and then, cells were stained with PI/annexin V and quantitatively analyzed the cell apoptotic rate. **(D)** After the same treatment described above, cell viability was measured using the MTT assay. **(E)** After the same treatment described above, caspase-3/-8 activities were assayed. Data are the mean ± SD of three independent experiments. **p < 0.01, compared with the combined treatment group after transfection of DR5 siRNA.

Given that we observed that alternol induces the upregulation of DR5 but not of DR4, the role of DRs in TRAIL-induced apoptosis was investigated using specific siRNA against DR5 and DR4. Transfection with DR5 and DR4 siRNA but not scramble siRNA significantly downregulated DR5 and DR4 expression, respectively (Figure 3A). We next tested whether silencing DR5 or DR4 expression affected the effect of alternol on TRAIL-induced apoptosis. We found that alternol was unable to potentiate TRAIL-induced apoptosis in Caki-1 cells after DR5 silencing (Figures 3B,C). However, DR4 siRNA or scramble siRNA transfection had minimal effect on the potentiation of TRAIL-induced apoptosis by alternol (Figures 3B,C). Meanwhile, the antiproliferative effects of the alternol, TRAIL or combined alternol, and TRAIL treatment were also reduced after transfection with DR5 siRNA but not with DR4 or scramble siRNA (Figure 3D). Moreover, the enhancement of caspase-3/-8 activities caused by cotreatment of alternol and TRAIL was abrogated by silencing of DR5 but not DR4 (Figure 3E). Overall, these findings suggest that DR5 plays a key role in TRAIL/alternol-induced apoptosis and that the enhancement of apoptosis by alternol is due to DR5 upregulation.

### Alternol Decreases Antiapoptotic Protein Expression and Increases Proapoptotic Protein Expression

**FIGURE 4:**
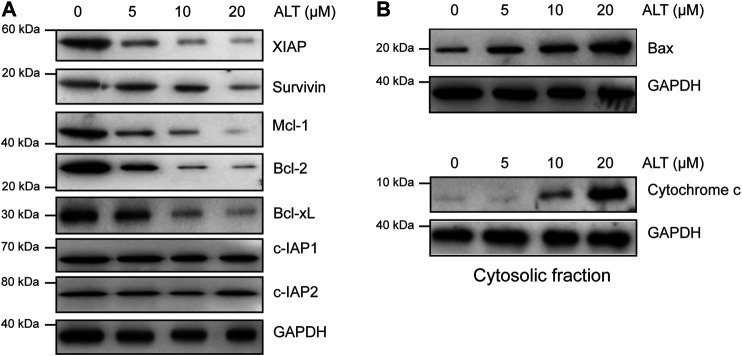
Effects of alternol on apoptotic protein expression. Caki-1 cells were treated with the indicated concentrations of alternol for 24 h. Whole-cell extracts were subjected to western blot assay using various antibodies. **(A)** The expression of antiapoptotic proteins. **(B)** The change of proapoptotic proteins after alternol treatment.

We then examined other mechanisms underlying the ability of alternol to enhance TRAIL-induced apoptosis. Various anti- and proapoptotic proteins are involved in TRAIL-induced apoptosis, so we examined whether alternol enhances TRAIL-induced apoptosis through modulating these proteins. We found that alternol inhibited XIAP, survivin, Bcl-2, Mcl-1, and Bcl-xl expression in a dose-dependent manner but had a minimal effect on c-IAP1, c-IAP2, and Bcl-xl expression (Figure 4A). We also found that alternol could induce the expression of the proapoptotic proteins Bax and the release of cytochrome c from mitochondria into cytosol in a dose-dependent manner (Figure 4B). Taken together, these results suggest that alternol downregulates antiapoptotic protein expression while upregulating proapoptotic protein levels. These two effects are additional mechanisms by which alternol potentiates TRAIL-induced apoptosis.

### Alternol Inhibited Akt Activation and Upregulated CHOP, which Relied on the JNK Activation

**FIGURE 5:**
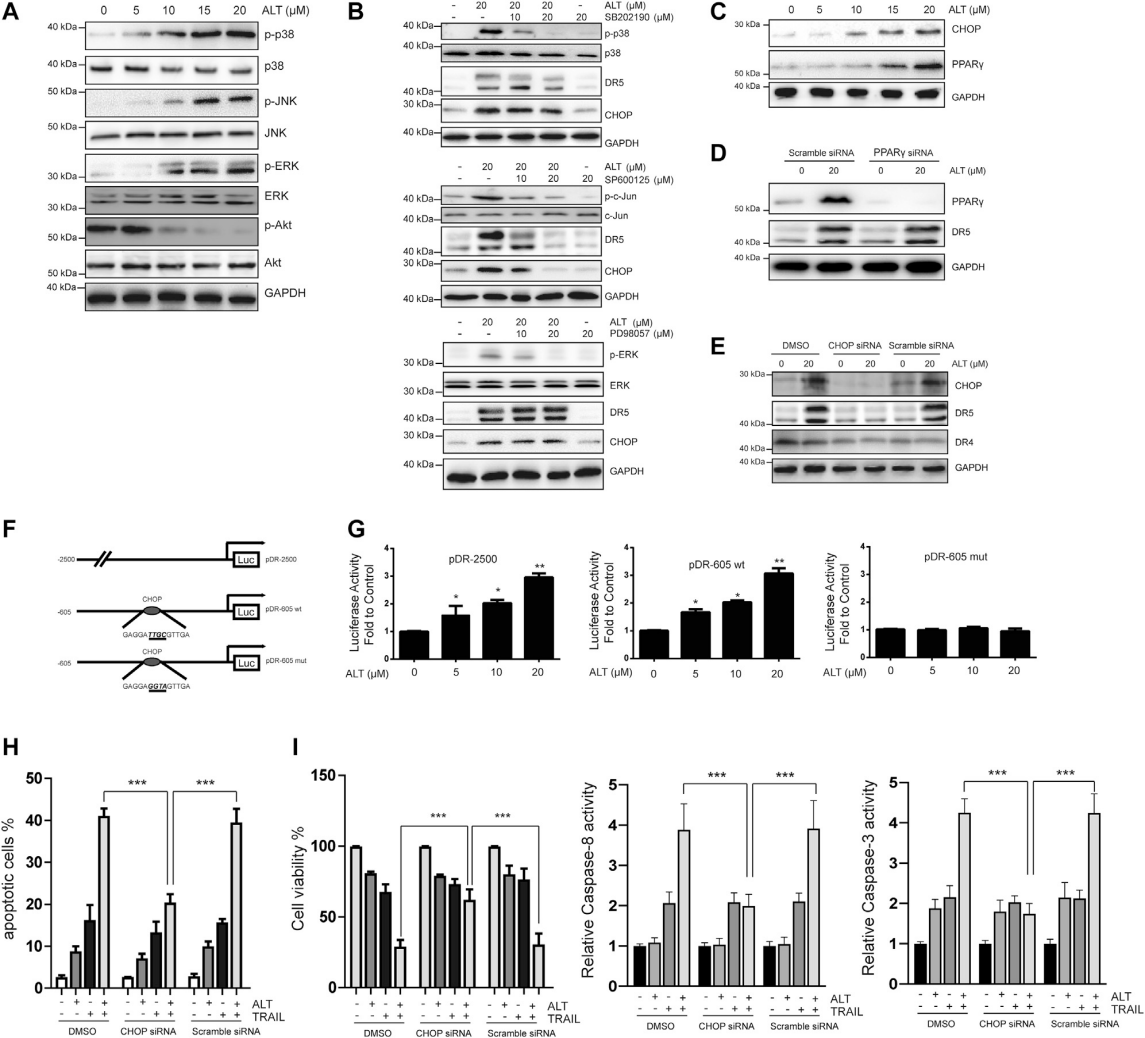
Upregulation of DR5 depends on CHOP. **(A,B,C)** Caki-1 cells were treated with various concentrations of alternol for 24 h. Total cellular extracts were subjected to western blot analysis with indicated antibodies. **(D)** Caki-1 cells were transfected with PPARγ siRNA or scramble siRNA for 24 h. Cells were then treated with 20 μM alternol for an addition 24 h, and total cellular extracts were subjected to western blot assay. **(E)** Caki-1 cells were transfected with CHOP siRNA or scramble siRNA for 24 h. Cells were then treated with 20 μM alternol for an addition 24 h, and total cellular extracts were subjected to western blot assay. **(F)** Schematic structures of the DR5 promoter constructs used to measure luciferase activity. Mutations were introduced into the CHOP consensus sites. **(G)**. Caki-1 cells were transfected with the reporter constructs, and lysates from cells that had been treated with various doses of alternol were assayed for luciferase activity. **(H)** Caki-1 cells were transfected with CHOP siRNA and scramble siRNA. Twenty-four h after the transfection, cells were treated alternol (20 μM), TRAIL (50 ng/ml), or their combination for an additional 24 h, and the apoptotic rate was determined by PI/annexin V staining. **(I)** After the treatment described above, cell viability was measured by MTT assay. **(J)** After the treatment described above, caspase-3/-8 activities were assayed. Data are the mean ± SD of three independent experiments. *p < 0.05; **p < 0.01; ***p < 0.001, compared with the untreated group.

Many reports have indicated that MAPK pathways modulate DR5 expression (Gupta et al., 2011). To investigate whether alternol activates MAPKs, cells were treated with various doses of alternol. Then, MAPK activation was assessed by western blot assays. As indicated in Figure 5A, all MAPKs were activated after the alternol treatment. Suppressed Akt activation is essential for TRAIL-induced apoptosis (Shoeb et al., 2013; Min et al., 2014). Therefore, we also tested whether alternol inhibited Akt activation. We observed that alternol suppressed Akt activation (Figure 5A). Then, specific inhibitors against p38, JNK, and ERK were used to examine which MAPK was accounted for the alternol-induced upregulation of DR5. Pretreating Caki-1 cells only with SP600125 (JNK inhibitor) inhibited the alternol-induced enhanced expression of DR5 in a dose-dependent manner (Figure 5B). Therefore, alternol induced DR5 upregulation via activation of the JNK pathway. We continued to investigate the mechanism behind DR5 upregulation. Various studies have indicated that DR5 can be upregulated by PPARγ ligands (Zou et al., 2007; Su et al., 2008). We then asked whether alternol affects PPARγ expression. However, we observed that alternol increased PPARγ expression in a dose-dependent manner (Figure 5C). However, silencing of PPARγ did not abrogate the upregulation of DR5 induced by alternol (Figure 5D). Many agents, including PPARγ agonists, induce DR5 upregulation through the expression of the transcription factor CHOP (Zang et al., 2009). Thus, we next studied whether alternol enhanced CHOP expression. Similar to PPARγ, we also found that alternol increased CHOP expression in a dose-dependent manner (Figure 5C). In contrast, it was found that the increased expression of DR5 was markedly abolished after CHOP was silenced, whereas the treatment with scrambled siRNA had no effect (Figure 5E).

Then, we asked whether CHOP transcriptional activity was vital for alternol-enhanced DR5 expression. After alternol treatment, we compared the activities of the following three DR5 promoters: a pDR-2500 construct, which harbored the DR5 promoter sequence (2,500 bp of the upstream region); a pDR-605wt construct, which contained the DR5 promoter sequence (605 bp of the upstream region); and a pDR-605mut construct, which contained the mutated CHOP binding site from the pDR-605wt plasmid (Figure 5F). As shown in Figure 5G, the pDR-2500 and pDR-605wt promoter activities were markedly increased by alternol incubation. However, the promoter activity of pDR-605mut was not significantly altered by alternol treatment. Therefore, alternol-triggered increased expression of DR5 is transcriptionally controlled by CHOP.

Finally, we examined whether silencing CHOP nullified alternol-sensitized, TRAIL-induced apoptosis. We observed that the effect of alternol on TRAIL-induced apoptosis was diminished in cells after CHOP was silenced, whereas the scramble siRNA treatment had minimal effects (Figure 5H). The alternol-mediated increase in TRAIL cytotoxicity was also abolished by downregulating CHOP expression (Figure 5I). Furthermore, the augment of caspase-3/-8 activities induced by alternol and TRAIL cotreatment was abrogated by silencing of CHOP as well (Figure 5J).

Therefore, the alternol-induced DR5 expression was dependent on CHOP expression.

### Alternol-Mediated DR5 Upregulation and TRAIL-Induced Apoptosis Are ROS Dependent

**FIGURE 6:**
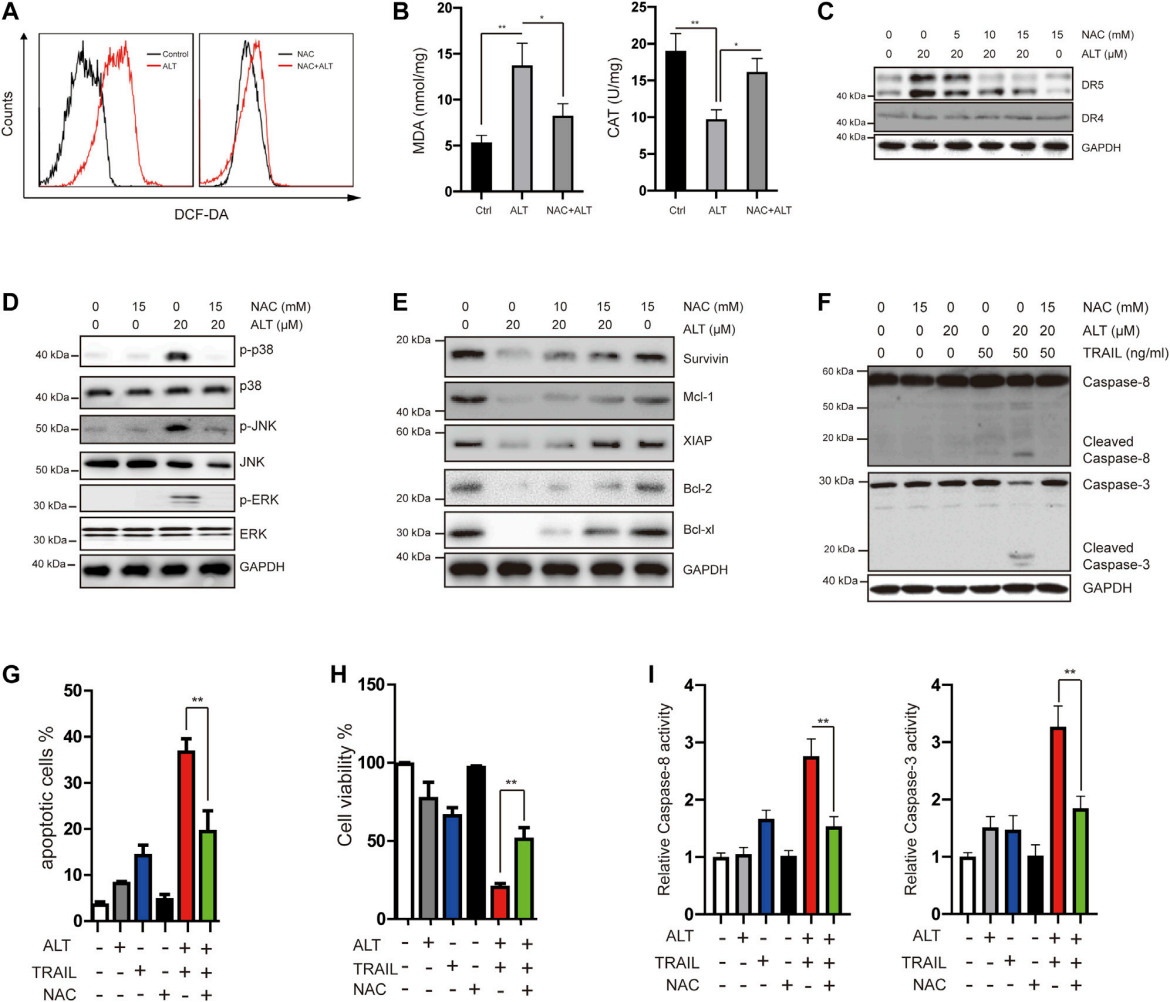
Alternol induces generation of ROS, and alternol-induced sensitization of TRAIL was mediated by ROS. **(A)** Caki-1 cells were labeled with DCF-DA and incubated with 20 μM alternol for 6 h **(left)**. Caki-1 cells were preexposed to NAC for 1 h, washed with PBS, labeled with DCF-DA, and treated with 20 μM alternol for 6 h **(right)**. ROS generation was measured by a flow cytometer. **(B)** Caki-1 cells were preexposed to NAC for 1 h, washed with PBS, and treated with 20 μM alternol for 6 h. MDA levels and CAT activity were measured. **(C,D,E,F)** Caki-1 cells were treated as indicated for 24 h, and then total cellular lysates were subjected to western blot analysis with indicated antibodies. **(G)** Caki-1 cells were treated with NAC (15 mM), alternol (20 μM), and TRAIL (50 ng/ml) as indicated for 24 h. The apoptotic cells were analyzed by PI/annexin V staining assay. **(H)** After the treatment described above, the cell viabilities were analyzed by MTT assay. **(I)** After the treatment described above, caspase-3/-8 activities were assayed. Data are the mean ± SD of three independent experiments. **p < 0.01, compared with the untreated group.

Many reports have implicated ROS in DR5 induction (Prasad et al., 2014; Trivedi et al., 2014). We investigated whether alternol exerts its effect through ROS. We treated Caki-1 cells with alternol and used dichlorofluorescein diacetate (DCF-DA) as a probe to assess the changes in ROS levels. We found that alternol generated ROS, which was abrogated by the ROS scavenger N-acetylcysteine (NAC) (Figure 6A). Other indicators of oxidative stress, such as MDA and CAT, were also measured. MDA levels were increased, whereas CAT activity was decreased after treatment with alternol (Figure 6B). Next, we investigated whether ROS generation is required for alternol-induced DR5 expression upregulation. We observed that NAC pretreatment in Caki-1 cells blocked alternol-induced DR5 expression in a dose-dependent manner (Figure 6C), which indicates that ROS production is essential for alternol-mediated upregulation of DR5 expression. Then, we studied whether ROS generation is essential for alternol-induced activation of MAPKs. NAC pretreatment in Caki-1 cells abolished the activation of MAPKs (Figure 6D).

Given that we observed that alternol treatment downregulates the expression of several antiapoptotic proteins, we next tested whether NAC abrogates this effect. As shown in Figure 6E, NAC treatment effectively abrogated alternol-inhibited survivin, Mcl-1, XIAP, Bcl-2, and Bcl-xl expression.

In the end, we asked whether ROS is required for alternol to potentiate TRAIL-induced apoptosis and anti-proliferation. We found that the NAC pretreatment noticeably interfered with the effect of alternol on TRAIL-induced caspase activation (Figure 6F). We also observed that NAC inhibited alternol-enhanced apoptotic and antiproliferative effects (Figures 6G). In addition, NAC also repressed alternol-induced increases caspase-3/-8 activities (Figure 6H).

Taken together, these findings underline the critical role of ROS in the effect of alternol on TRAIL treatment.

### Evaluation of the Combined Alternol and TRAIL Effects in a Xenograft Model

**FIGURE 7:**
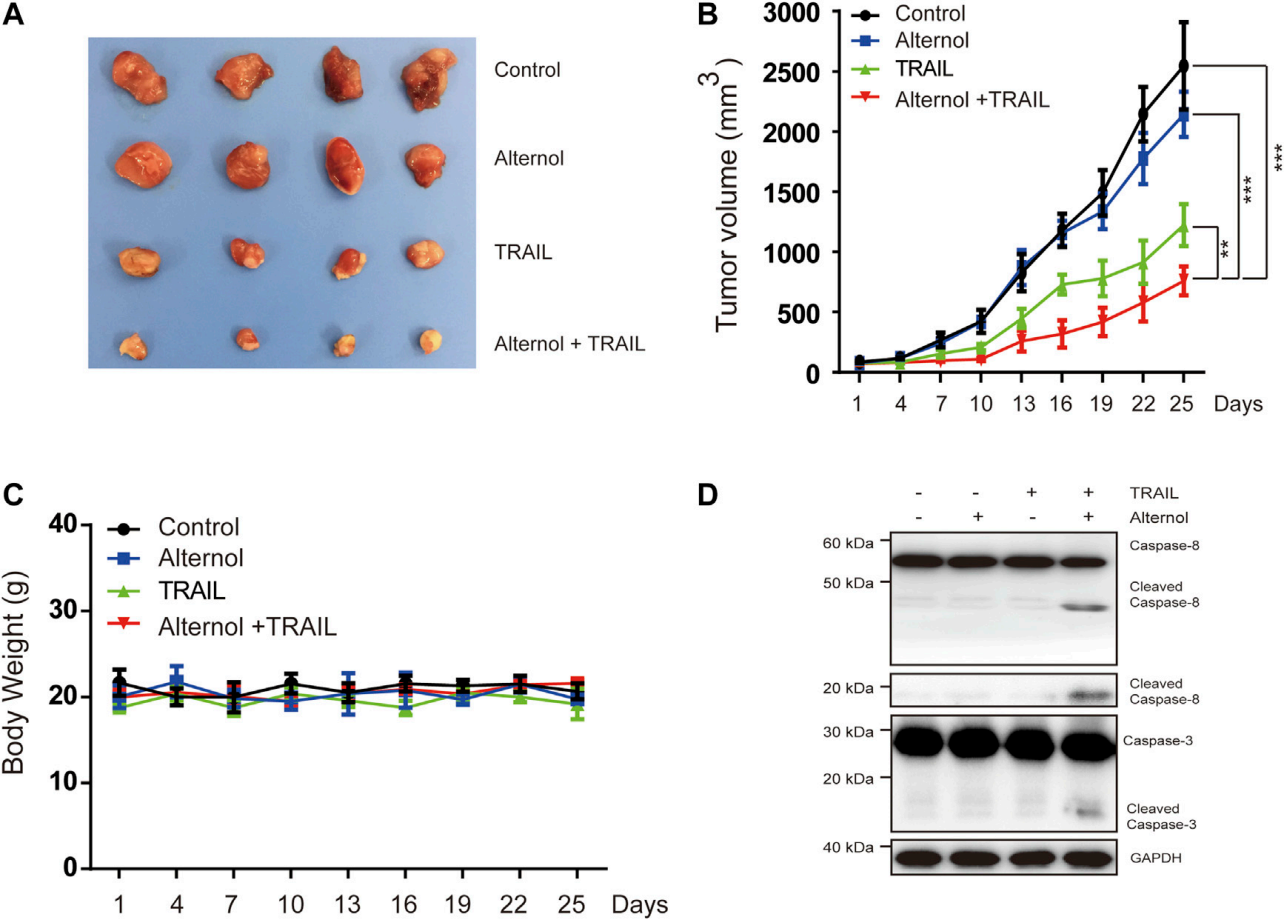
Effects of the combination therapy on the mouse RCC xenograft model. Caki-1 tumor-bearing nude BALB/c mice were treated with a vehicle control, alternol, TRAIL, or combination. **(A)** Posttreatment changes in tumor volumes. **(B)** Posttreatment changes in tumor size. **(C)** Body weight changes in each group. **(D)** The indicated proteins were determined by western blot in tumors. Data are the mean ± SD of three independent experiments. *p < 0.05; **p < 0.01; *p* < 0.001, compared with control.

Finally, we tested the *in vivo* antitumor effects of alternol/TRAIL cotreatment in a nude xenograft model. To generate the mouse model, Caki-1 cells were subcutaneously inoculated into nude mice. After the tumor diameters were found to be approximately 3–5 mm, the mice were matched by tumor size and divided into the following groups: the vehicle-treated, alternol-treated, TRAIL-treated, and alternol/TRAIL combination-treated groups. As shown in Figures 7A,B, tumor growth was significantly inhibited in the alternol/TRAIL combination group compared with the other groups. During the tumor xenograft experiments, body weight was not significantly different among the control and treatment groups (Figure 7C). Moreover, the effects of alternol/TRAIL cotreatment on the expression of proteins associated with apoptosis, such as caspase-3 and -8, were the same as those *in vitro* (Figure 7D).

## Discussion

To overcome TRAIL resistance in multiple carcinomas, a TRAIL sensitizer is needed for more effective TRAIL-based therapeutic strategies. In the present study, we demonstrated that alternol could work as a sensitizer to TRAIL in RCC cells. We found alternol enhances TRAIL-induced apoptosis in RCC through a variety of mechanisms, including the dependence of DR5 expression upregulation on the transcription factor CHOP, ROS generation, and antiapoptotic protein suppression.

Plenty of studies reported that alternol exerted antitumor effects via various mechanisms (Liu et al., 2020). In the current study, we found that alternol enhances the effects of TRAIL by inducing DR5 but not DR4 expression. The critical role of DR5 was confirmed, given that RNAi-mediated DR5 silencing inhibited apoptosis. Until now, various mechanisms have been identified to modulate the expression of DR5. For instance, three binding sites of p53 have been found in the DR5 promoter region (Wu et al., 1997). As an endoplasmic reticulum (ER) stress-induced transcriptional factor, CHOP has also been found playing critical roles on DR5 expression (Yamaguchi and Wang, 2004). It has also been documented that PPARγ, a member of the nuclear receptor family of transcription factors, was also able to regulate the expression of DR5 (Zou et al., 2007; Su et al., 2008). In our scenario, however, p53, CHOP, and PPARγ have all been found upregulated after the treatment of alternol. However, RNAi results suggested that alternol-induced expression of DR5 depend on the upregulation of CHOP. Interestingly, it was also found that the upregulation of DR5 was decreased but not fully inhibited in p53 knockout cells. According to previous studies, p53 is involved in the upregulation of CHOP (Sicari et al., 2019). In our scenario, we hypothesized that depletion of p53 attenuated the expression of CHOP and further decreased the expression of DR5. Considering that p53 status is mutated in nearly half of cancers, p53-independent upregulation of DR5 by alternol seems attractive.

Many studies have indicated that the activation of MAPKs, such as JNK or ERK, is required for DR5 expression induction (Sung et al., 2010; Gupta et al., 2013). In our study, we observed the activation of these kinases after the alternol treatment. Our findings are similar to a previous study which also found that alternol could induce activation of MAPKs in osteosarcoma (Zuo et al., 2017). Using specific inhibitors, we found that JNK activation is responsible for the upregulation of DR5 and CHOP expression (Figure 5B). This finding is consistent with previous studies showing JNK-dependent CHOP induction of DR5 (Gopalan et al., 2013; Wu et al., 2017). ROS are vital signaling molecules that affect various cellular responses.

ROS triggers a variety of signaling pathways such as MAPKs that lead to cell differentiation, growth, migration, or death. In accordance with previous reports, we demonstrated that alternol noticeably increased ROS production, which was associated with alternol-sensitized, TRAIL-induced apoptosis and reduced cell viability (Zuo et al., 2017). To further investigate the role of ROS, the ROS scavenger NAC was used and reversed the alternol-mediated activation of MAPKs (Figure 6D). Quenching of ROS also abrogated the effect of alternol on the upregulation of DR5 and TRAIL-induced apoptosis. Thus, all these findings implicate the critical role of ROS in the function of alternol. Our results are consistent with earlier studies on ROS-enhanced TRAIL activity (Gupta et al., 2011; Prasad et al., 2011; Gupta et al., 2013; Min et al., 2014).

Alternol enhanced TRAIL-induced apoptosis involved both the extrinsic and the intrinsic pathways, as evidenced by a robust activation of caspase-3 and -8. This finding suggested that alternol/TRAIL triggers the extrinsic apoptotic pathway in Caki-1 cells. RCC cells are generally insensitive to chemotherapeutic agents because of the blockage of intrinsic apoptotic pathway (Diamond et al., 2015). Thus, combination of alternol and TRAIL might provide an alternative strategy to kill RCC cells. According to previous studies, alternol-induced apoptosis is mechanistically caused by disturbance of anti- and proapoptotic proteins (Liu et al., 2020). Thus, we also examined the effects of alternol on the expression of apoptosis-related proteins. It was found that alternol inhibited the expression of several antiapoptotic proteins, such as XIAP, Mcl-1 Bcl-2, Bcl-xl, and survivin, but had minimal effect on c-IAP1 and c-IAP2 levels. Among these affected proteins, XIAP is a well-documented potent inhibitor of caspase-3 and has previously been reported to confer TRAIL resistance (Mohr et al., 2010; Yu et al., 2013). Downregulated survivin or Mcl-1 expression is also related to increased TRAIL-induced apoptosis (Min et al., 2014; Trivedi et al., 2014; Hwang et al., 2015). Notably, inhibiting Mcl-1 with other agents also sensitizes TRAIL-resistant Caki-1 cells to TRAIL-induced apoptosis (Min et al., 2014; Han et al., 2016). Therefore, Mcl-1 may represent a good target for TRAIL-based therapies in renal carcinomas. Although the mechanism by which alternol affects the expression of those proteins was not fully investigated in this study, several possible mechanisms may account for this effect. First, Mcl-1 is a well-documented, short-lived protein (Thomas et al., 2010). PI3K/Akt activation may increase Mcl-1 stability (Derouet et al., 2004; Morton et al., 2015). Therefore, p-Akt inhibition by alternol may also contribute to Mcl-1 downregulation. Second, we found that alternol induced PPARγ expression, which has been implicated in the downregulation of antiapoptotic proteins (Pellerito et al., 2010). Third, we found that ROS regulated the downregulation of these proteins. According to previous studies, ROS positively affects proteasome activity. For example, ROS increases proteasome activity in skeletal muscle myotubes and lens epithelial cells (Shang et al., 1997; Li et al., 2003). ROS increased proteasome activity and downregulated the levels of survival proteins in multiple studies (Gupta et al., 2011; Prasad et al., 2011; Min et al., 2014). However, whether alternol downregulates those proteins through modulating proteasome activity requires further investigation.

Our study indicates that alternol has potential clinical relevance in combination with TRAIL therapy. The combination of alternol with TRAIL inhibited tumor volume in an *in vivo* mouse xenograft model and increased cleaved caspase-3 and -8 expression in tumor tissues. Although we did not perform any pharmacokinetic experiments, the dose of alternol that we used (20 mg/kg) sensitizes RCCs to TRAIL *in vivo* without obvious cytotoxicity, indicating that alternol may be provided at a clinically efficacious and safe dose.

In the present study, we observed that alternol increased TRAIL-induced apoptosis via various mechanisms. In conclusion, the use of TRAIL in combination with alternol might constitute an effective therapeutic strategy for the treatment of some TRAIL-insensitive renal carcinomas.

## Data Availability

The original contributions presented in the study are included in the article/Supplementary Material; further inquiries can be directed to the corresponding authors.
