# Supplementary figures and images for: Alternol Sensitizes Renal Carcinoma Cells to TRAIL-Induced Apoptosis
Source: Front Pharmacol. 2021 Mar 25;12:560903. doi: 10.3389/fphar.2021.560903 (PMC8026879; doi:10.3389/fphar.2021.560903)

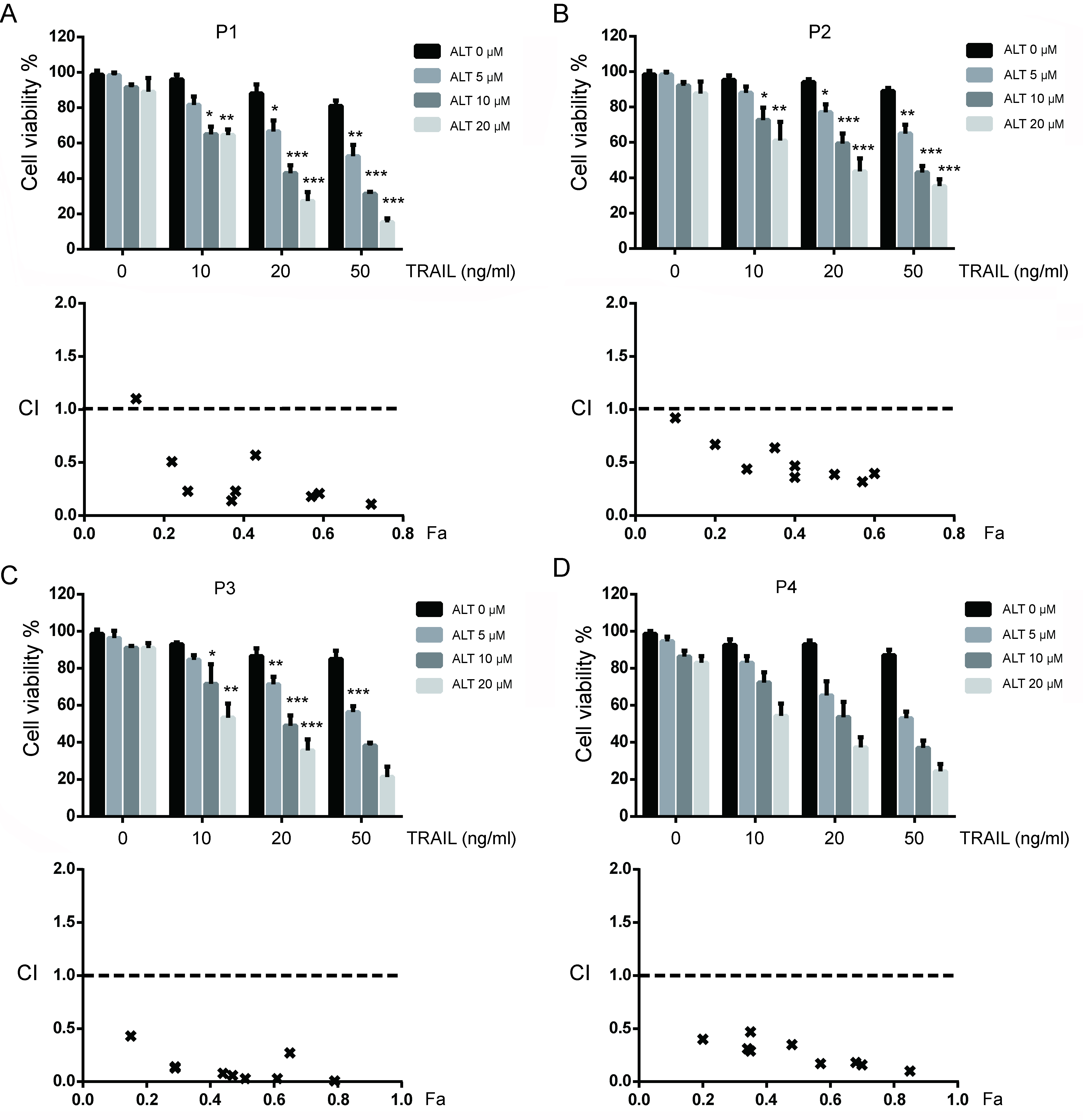

Supplement: Supplementary file 1 [file image1.tif]

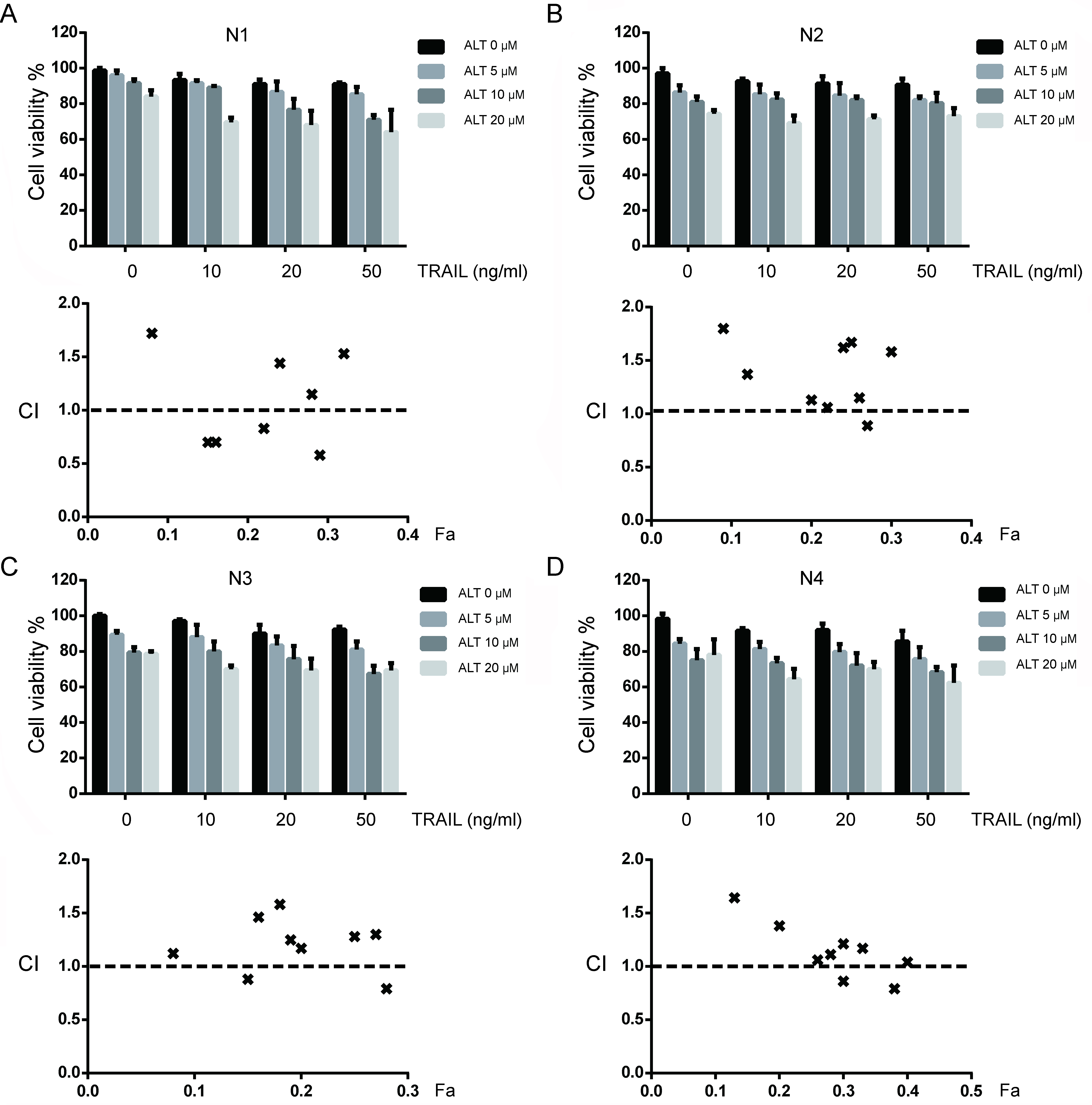

Supplement: Supplementary file 2 [file image2.tif]
